# Edible Cactus Powder-Enriched Cassava and Rice Flours as Fiber-Enriched Plant-Based Ingredients: Physicochemical, Rheological, Freeze–Thaw Properties and Starch Digestibility

**DOI:** 10.3390/foods15214005

**Published:** 2026-11-09

**Authors:** Tipaukson Chaikwang, Hua Li, Kriangsuk Boontiang, Sirithon Siriamornpun

**Affiliations:** 1 Department of Food Technology and Nutrition, Faculty of Technology, Mahasarakham University, Kantarawichai District, Maha Sarakham 44150, Thailand; 67010853502@msu.ac.th; 2 Department of Cuisine and Nutrition, Yangzhou University, Yangzhou 225127, China; 3 Key Laboratory of Chinese Cuisine Intangible Cultural Heritage Technology Inheritance, Ministry of Culture and Tourism, Yangzhou 225127, China; 4 Department of Agricultural Technology, Faculty of Technology, Mahasarakham University, Maha Sarakham 44150, Thailand; kriangsuk.b@msu.ac.th; 5 Research Unit of Thai Food Innovation (TFI), Mahasarakham University, Kantarawichai, Maha Sarakham 44150, Thailand

**Keywords:** *Opuntia ficus-indica* (L.) Mill., gluten-free, plant-based food, fiber content, mucilage, functional ingredient

## Abstract

Understanding how plant-based ingredients influence the functional properties of starch-based flours is important for their potential applications in gluten-free food development. This study investigated the incorporation of edible cactus powder (CP) into cassava flour (CF) and rice flour (RF) at 5%, 10%, and 15% and evaluated its effects on their physicochemical, structural, pasting, rheological, freeze–thaw, and in vitro starch digestibility properties. CP incorporation increased crude fiber content from 0.32 to 3.41 g/100 g in CF and from 0.73 to 2.55 g/100 g in RF at 15% CP, while increasing the measured levels of bioactive compounds and antioxidant activity. SEM, FTIR, and XRD analyses indicated changes in the morphological, spectral, and diffraction characteristics of the flour blends. CP generally reduced the viscoelastic moduli (G′ and G″), apparent viscosity, and most pasting viscosity parameters, while reducing freeze–thaw syneresis under the experimental conditions. Furthermore, CP-fortified flour blends generally exhibited lower rapidly digestible starch (RDS) and higher slowly digestible starch (SDS) fractions, providing preliminary insights into their in vitro starch digestibility characteristics. Overall, the findings provide preliminary insights into the physicochemical and functional characteristics of CP-fortified flour blends and a basis for further investigation of their potential applications in gluten-free food formulations.

## 1. Introduction

Cassava (*Manihot esculenta* Crantz.) and rice (*Oryza sativa* L.) are major economic crops that are widely cultivated and utilized in Thailand. Cassava flour (CF) and rice flour (RF) are important starch-based raw materials that contribute substantially to the agricultural, economic, and food industries of Thailand. Cassava is highly adaptable and can grow well under dry conditions and irregular rainfall, making it a suitable crop for production in areas with limited water availability [1]. Rice, meanwhile, is a major staple crop that is extensively cultivated throughout Thailand [2]. The widespread production of these crops provides abundant raw materials for the manufacture of cassava and rice flours, which can be used in a wide range of food applications. In terms of chemical composition, CF and RF are predominantly composed of carbohydrates, while containing relatively low levels of protein, lipid, and dietary fiber. CF contains approximately 0.06–0.75% protein, 0.01–1.20% lipid, and 0.11–1.90% dietary fiber [3]. RF, in comparison, contains approximately 0.91–1.66% dietary fiber and 1.13% lipid [4]. These compositional characteristics indicate certain nutritional limitations of both flours, particularly in terms of dietary fiber and bioactive compounds. Nevertheless, CF and RF remain widely used in food product development because of their desirable functional properties, including thickening capacity, gel-forming ability, and the ability to modify the texture of food products [5,6]. These functional properties have contributed to their wide application in various food products, such as bakery products, noodles, snacks, sauces, and other gluten-free products. Therefore, there is a need to explore approaches for improving the nutritional value of CF- and RF-based foods while maintaining their desirable functional properties. The incorporation of nutrient-rich plant-based ingredients, particularly those providing dietary fiber and bioactive compounds, represents a potential strategy for developing nutritionally enhanced flour systems and flour-based foods.

Edible cactus cladodes of *Opuntia ficus-indica* (L.) Mill. are a promising plant-based ingredient because they contain dietary fiber, minerals, phenolic compounds, flavonoids, and other phytochemicals [7,8]. In addition, cactus cladodes are rich in dietary fiber and contain pectin and mucilage, which may influence water distribution and the physicochemical behavior of food matrices [9,10]. These characteristics have encouraged the incorporation of cactus-derived ingredients into different food products. For example, the addition of cactus cladode to pasta formulations has been reported to increase dietary fiber and bioactive compound contents, whereas cactus-derived gum has been used in cookies to enhance soluble dietary fiber while maintaining consumer acceptability [11,12]. Such findings suggest that cactus-derived ingredients may provide not only nutritional benefits but also functional effects when incorporated into food matrices. Previous studies have demonstrated that cactus cladode can modify the properties of flour- and dough-based systems. Ayadi et al. [13] reported that incorporating cactus cladode powder into wheat flour at 5–20% altered dough strength, energy, cohesiveness, adhesiveness, and hardness while reducing extensibility. Similarly, Campos-Pastelin et al. [14] found that incorporating cactus cladode powder into bread formulations at 3–10% modified dough structure and increased protein and crude fiber contents, accompanied by changes in pH and water activity. In addition to these physicochemical effects, cactus powder incorporation has been associated with changes in bioactive compound contents. Msaddak et al. [15] reported an increase in total phenolic content (TPC) from 0.90 to 9.36 mg GAE/100 g following the partial replacement of wheat flour with cactus cladode powder at levels of 2.5–10%. Likewise, Aparicio-Ortuño et al. [16] reported TPC values ranging from approximately 100 to 500 mg GAE/100 g in food products containing 10–25% cactus cladode powder. Collectively, these studies suggest that cactus-derived ingredients can influence the physicochemical properties and phenolic composition of flour-based food products. However, previous investigations have primarily focused on wheat-based systems or formulated food products, where the observed effects may also be influenced by gluten development and other formulation components. In contrast, limited information is available regarding the effects of cactus powder incorporation on cassava and rice flour systems, particularly how these effects may differ between flour matrices with distinct compositional and structural characteristics.

Cassava and rice flours differ in their starch composition and structural characteristics, which may influence their responses to cactus powder (CP) incorporation. Although CP contains fiber-related components, including mucilage, that may affect water retention and flow behavior [3,10,17], its effects on the chemical composition, structural characteristics, pasting and rheological properties, freeze–thaw stability, and in vitro starch digestibility of these flour systems have not been comprehensively investigated. Importantly, a comparative evaluation of CF and RF under the same CP incorporation levels may provide a more comprehensive understanding of how different flour matrices respond to CP and how their physicochemical and functional properties are modified. Such information is valuable for establishing a scientific basis for future research on CP-containing gluten-free food formulations, particularly those requiring specific flow characteristics and freeze–thaw stability.

Therefore, this study aimed to investigate the effects of incorporating edible cactus powder (CP) into cassava flour (CF) and rice flour (RF) systems at different replacement levels, with particular emphasis on changes in their chemical composition and functional properties. The chemical composition, structural characteristics, pasting and rheological properties, freeze–thaw stability, and in vitro starch digestibility of both flour systems were systematically evaluated. By comparing the responses of CF and RF to CP incorporation, this study seeks to provide a more comprehensive understanding of the physicochemical and functional characteristics of CP-fortified flour blends. Importantly, the findings are expected to provide fundamental information that may support the selection of suitable flour systems and guide future formulation and product development studies involving CP, particularly for gluten-free foods requiring specific flow, water-retention, and freeze–thaw properties.

## 2. Materials and Methods

### 2.1. Materials and Reagents

Edible cladodes of *Opuntia ficus-indica* (L.) Mill. were obtained from Maha Sarakham Province, Thailand (16.1872° N, 103.3045° E). Rice flour (RF) was obtained from Thai Flour Industry Co., Ltd. (Bangkok, Thailand), while cassava flour (CF) was obtained from E.T.C. Eaib Tong Chan Co., Ltd. (Nonthaburi, Thailand). All chemicals were purchased from Sigma-Aldrich (St. Louis, MO, USA) and were used for the analysis of bioactive compounds, antioxidant activity, and in vitro digestion.

### 2.2. Cladode Sample Preparation

Edible cactus cladodes were thoroughly washed with distilled water, freeze-dried, ground, and passed through a 50-mesh sieve to obtain cactus powder (CP). CP partially replaced cassava flour (CF) or rice flour (RF) at 5%, 10%, and 15% (*w*/*w*), corresponding to flour-to-CP ratios of 95:5, 90:10, and 85:15, respectively. The blends were stored at −20 °C until analysis.

### 2.3. Color Parameter

Color values of the samples were measured using a CR-400/CR-410 Chroma Meter (Konica Minolta Sensing Inc., Osaka, Japan). Measurements were performed in triplicate, and the results were expressed in the CIELAB color space as (L*), (a*), and (b*). Chroma and hue angle were calculated from the measured (a*) and (b*) values using the following Equations (1) and (2):

(1) $$\mathrm{Chroma}=\sqrt{{\left(a^{*}\right)}^{2}+{\left(b^{*}\right)}^{2}}$$

(2) $$\mathrm{Hue}\;\mathrm{angle}=180{}^{\circ}+\tan^{-1}\left(\frac{b*}{a*}\right)$$

Hue angle values were expressed in degrees within the range of 0–360°

### 2.4. Chemical Composition Analysis

The proximate composition of the samples was determined according to the method described by Boonarsa et al. [18], with the moisture, ash, lipid, protein, and crude fiber content analyzed using the corresponding AOAC methods (925.10, 900.02, 920.177, 920.176, and 978.10, respectively). A nitrogen-to-protein conversion factor of 6.25 was used for protein calculation. Carbohydrate content was calculated by difference as follows:

Carbohydrate (%) = 100 − [moisture (%) + ash (%) + lipid (%) + protein (%) + crude fiber content (%)](3)

All compositional results were expressed as g/100 g sample on an as-is basis.

### 2.5. Antioxidant Activities and Bioactive Compounds

#### 2.5.1. Extraction for Bioactive Compounds and Antioxidant Activity

The samples were extracted according to the methods described by Wan Majid et al. and Kaewseejan et al. [19,20], with slight modifications. Briefly, 1.0 g of each sample was weighed and extracted with 20 mL of 95% ethanol using a shaker at room temperature for 18 h. The resulting extract was collected and stored in an amber bottle for subsequent analysis of bioactive compounds.

#### 2.5.2. Total Phenol Content (TPC) and Total Flavonoid Content (TFC)

The TPC and TFC were determined according to the method of Raksakantong et al. [21,22] with minor modifications. For TPC determination, 0.2 mL of the sample extract was mixed with 0.5 mL of 10% Folin–Ciocalteu reagent, followed by the addition of 2.25 mL of 7% Na_2_CO_3_ solution. The mixture was allowed to react at room temperature for 90 min, and the absorbance was measured at 725 nm using a UV–visible spectrophotometer (UV-1700, Shimadzu, Tokyo, Japan). TPC was quantified using a gallic acid calibration curve and expressed as milligrams of gallic acid equivalents per 100 g of dry weight (mg GAE/100 g DW). For TFC determination, 1.0 mL of the sample extract was mixed with 0.3 mL of 5% (*w*/*v*) sodium nitrite (NaNO_2_) solution and incubated in the dark for 5 min. Subsequently, 0.5 mL of 2% (*w*/*v*) aluminum chloride hexahydrate (AlCl_3_·6H_2_O) solution was added, and the mixture was allowed to stand for 6 min before adding 0.5 mL of 1 M sodium hydroxide (NaOH) solution. The absorbance was measured at 510 nm using the same spectrophotometer. TFC was quantified using a quercetin calibration curve and expressed as milligrams of quercetin equivalents per 100 g of dry weight (mg QE/100 g DW).

#### 2.5.3. The Ferric Reducing Antioxidant Power (FRAP) Assay and 2,2-Diphenyl-1-Picrylhydrazyl (DPPH) Radical Scavenging Assay

The ferric reducing antioxidant power (FRAP) assay was performed using the original method of Benzie and Strain [23], with minor modifications as described by Butsat et al. [24]. Briefly, 0.5 mL of the sample extract was mixed with 4.5 mL of freshly prepared FRAP reagent. The reagent was prepared by mixing 0.3 M acetate buffer (pH 3.6), 10 mM 2,4,6-tripyridyl-s-triazine (TPTZ) dissolved in 40 mM HCl, and 20 mM FeCl_3_ dissolved in distilled water at a volume ratio of 10:1:1. The mixture was incubated at 37 °C for 4 min, and the absorbance was then measured at 593 nm using a spectrophotometer. The results were expressed as mg ferrous sulfate equivalents per 100 g dry weight (mg FeSO_4_/100 g DW). The 2,2-diphenyl-1-picrylhydrazyl (DPPH) radical scavenging assay was performed using the original method of Brand-Williams et al. [25], with minor modifications as described by Boonarsa et al. [18], Briefly, 0.5 mL of the sample extract was mixed with 2.0 mL of 0.06 mM DPPH solution. The mixture was vortexed and incubated in the dark at room temperature for 30 min. The absorbance was then measured at 517 nm using a spectrophotometer. The results were expressed as mg L-ascorbic acid equivalents per 100 g dry weight (mg AA/100 g DW).

### 2.6. Structural Characterization

Microstructural observations were performed using a scanning electron microscope (SEM) (HITACHI TM4000Plus, Ibaraki, Japan). Samples were mounted on aluminum stubs, sputter-coated with gold, and imaged at an accelerating voltage of 10 kV in backscattered electron (BSE) mode at three magnifications (300×, 500×, and 1000×). One image was obtained at each magnification for each sample. Fourier transform infrared spectroscopy (FTIR) analysis of the samples was performed using an INVENIO S/LUMOS II FT-IR spectrometer (Bruker, Switzerland) over the spectral range of 400–4000 cm^−1^. X-ray diffraction (XRD) analysis was carried out using a D8 Advance X-ray diffractometer (Bruker, Germany) operated at 40 kV and 40 mA. Diffraction patterns were recorded over a 2θ range of 5–40° at a scanning rate of 2°/min.

### 2.7. Pasting Properties Analysis

Pasting properties were determined using a Rapid Visco Analyzer (RVA-4, Newport Scientific, Warriewood, Australia) according to the method of Charoenrein and Preechatham-Mawong [26] with slight modifications. Briefly, 3 g of flour sample was dispersed in 24.50 g of distilled water and stirred for 20 s in an aluminum canister before analysis. The sample was initially held at 50 °C for 1 min before being heated to 95 °C at a rate of 15 °C/min. The sample was then held at 95 °C for 3 min, cooled to 50 °C at a rate of 11.25 °C/min, and held at 50 °C for a further 2 min. The total analysis time was 13 min per sample. The results were expressed as the mean of triplicate measurements.

### 2.8. Rheological Analysis

Rheological measurements were performed in triplicate using gelatinized flour pastes obtained from three separate RVA runs for each formulation (Section 2.7). Following the RVA heating and cooling cycle, the pastes were transferred from the canisters and cooled to 25 °C before measurement. The rheological properties were evaluated using a HAAKE MARS 40 rheometer (Thermo Fisher Scientific, Karlsruhe, Germany) equipped with a 35 mm parallel-plate geometry (P35/Ti). The measurements were conducted at 25 ± 0.1 °C using a gap of 1 mm. After loading the sample between the parallel plates, excess material was carefully trimmed from the plate edges, and the sample was allowed to rest for 5 min at 25 °C to minimize residual stresses induced during sample loading before measurement. Frequency sweep measurements were performed according to Guo et al. [27], with slight modifications, over a frequency range of 0.1–10 Hz at a constant strain of 1%, which was within the linear viscoelastic region. The storage modulus (G′) and loss modulus (G″) were recorded as functions of frequency. Flow behavior was determined according to Ranathunga et al. [28], with slight modifications, by applying shear rates ranging from 0.1 to 100 s^−1^. The apparent viscosity (η) was measured at each shear rate, and the resulting flow behavior was evaluated based on the relationship between apparent viscosity and shear rate. Data acquisition and processing were performed using HAAKE RheoWin Data Manager 4 software (Thermo Fisher Scientific, Karlsruhe, Germany).

### 2.9. Freeze–Thaw Stability Analysis

The freeze–thaw stability of the flour gels was evaluated according to the method of Wu et al. [29], with slight modifications. The gelatinized flour gels obtained after the RVA analysis were allowed to stand for 5 min and then transferred into pre-weighed centrifuge tubes. The weight of each tube containing the gel was recorded to determine the initial gel weight. The samples were frozen at −18 °C for 24 h and subsequently thawed at 25 °C for 2 h. The freeze–thaw procedure was repeated for five cycles, and syneresis was determined after the first, third, and fifth cycles. At each designated cycle, the thawed samples were centrifuged at 4000 rpm for 20 min. The separated supernatant was carefully removed and weighed. Syneresis was expressed as the percentage of the weight of the separated liquid relative to the initial weight of the gelatinized gel, calculated using the following equation:

Syneresis (%) = [(M_2_ − M_3_)/(M_2_ − M_1_)] × 100 (4)

where M_1_ (g) is the weight of the centrifuge tube, M_2_ (g) is the weight of the centrifuge tube containing the starch paste before centrifugation, and M_3_ (g) is the weight of the centrifuge tube containing the starch paste after removal of the supernatant.

### 2.10. Analysis of In Vitro Starch Digestibility

The contents of rapidly digestible starch (RDS), slowly digestible starch (SDS), and resistant starch (RS) were subsequently determined following the method of Englyst et al. [30] and Seephua et al. [31], with modifications. Reducing sugars released during enzymatic hydrolysis were determined using the DNS method with glucose as the calibration standard. Briefly, 200 mg of uncooked sample was mixed with 15 mL of sodium acetate buffer (0.2 mol/L, pH 5.2). The mixture was then incubated at 37 °C with shaking at 160 rpm for 5 min. Subsequently, 5 mL of α-amylase (290 U/mL) and 5 mL of amyloglucosidase (15 U/mL) were added, and the mixture was further incubated at 37 °C with continuous shaking at 160 rpm. Aliquots (1 mL) were collected at 0, 20 and 120 min of hydrolysis and mixed with 4 mL of 66% aqueous ethanol to terminate the enzymatic reaction. The mixtures were then centrifuged at 6000 rpm for 10 min using an Allegra X-30R centrifuge (Beckman Coulter, Brea, CA, USA). The reducing sugar content in the supernatant was determined using the DNS (3,5-dinitrosalicylic acid) method with glucose as the calibration standard and expressed as glucose equivalents. Total starch content was determined using a commercial starch assay kit (BC0700; Beijing Solarbio Science & Technology Co., Ltd., Beijing, China). The assay is based on the anthrone-sulfuric acid colorimetric method. The total starch content was expressed as g/100 g sample and was used as TS in the calculation of the starch digestibility fractions. The reducing sugar contents were converted to g/100 g sample before calculating the starch digestibility fractions. RDS, SDS, and RS were calculated using Equations (5)–(7):

RDS (%) = [(G_20_ − G_0_) × 0.9/TS] × 100 (5)

SDS (%) = [(G_120_ − G_20_) × 0.9/TS] × 100 (6)

RS (%) = 100 − RDS − SDS(7)

where G_0_, G_20_, and G_120_ represent the reducing sugar contents, expressed as glucose equivalents (g/100 g sample), at 0, 20, and 120 min of hydrolysis, respectively; 0.9 is the conversion factor from glucose to starch; and TS represents the total starch content (g/100 g sample) of the corresponding formulation. RDS, SDS, and RS are expressed as percentages of total starch (% TS).

### 2.11. Statistical Analysis

Each formulation was prepared independently in triplicate (*n* = 3), and quantitative results are presented as mean ± standard deviation (SD). Data normality was assessed using the Shapiro–Wilk test. Statistical analyses were performed using IBM SPSS Statistics version 17 (IBM Corp., Armonk, NY, USA) and Origin 2022 (OriginLab Corporation, Northampton, MA, USA). One-way analysis of variance (ANOVA), followed by Duncan’s multiple range test, was used to compare the means across all nine sample groups, including CP, the unfortified CF and RF controls, and the CF and RF formulations containing 5%, 10%, and 15% CP. This approach allowed comparisons among all formulations within a single statistical framework. Differences were considered statistically significant at *p* < 0.05.

## 3. Results and Discussion

### 3.1. Color

**Figure 1:**
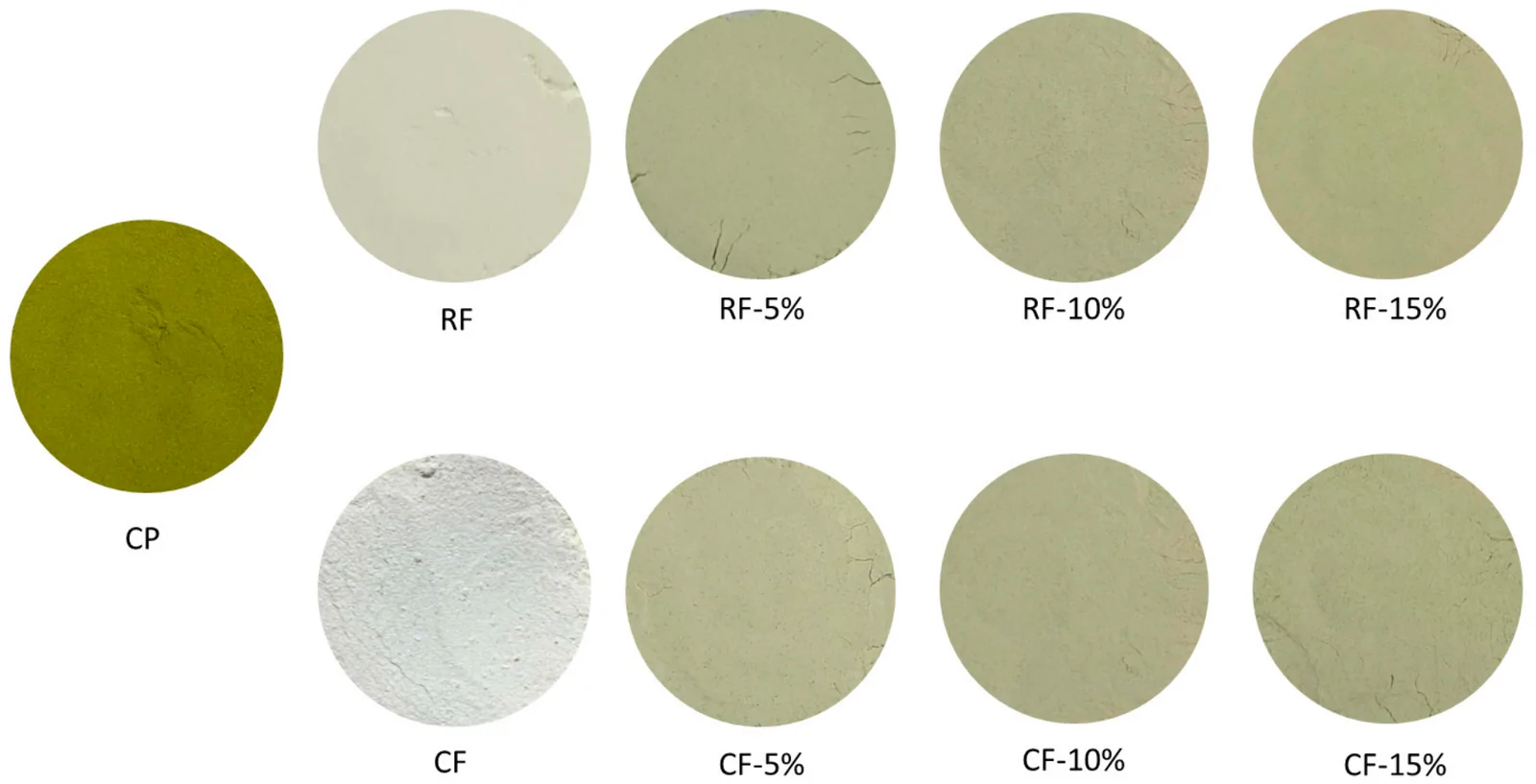
Appearance of rice flour and cassava flour supplemented with different concentrations of edible cactus powder. CP, edible cactus powder; RF, rice flour; CF, cassava flour. The values of 5%, 10%, and 15% indicate the concentrations of edible cactus powder added to the flour samples (*w*/*w*).

Color parameters are important attributes for evaluating the visual appearance of food products, as they influence product quality after processing and consumer acceptance [32]. Table 1 and Figure 1 present the color parameters of CF and RF fortified with CP at levels of 5%, 10%, and 15%. The results showed that increasing the concentration of CP affected the color parameters of both flour systems (*p* < 0.05). As the level of edible cactus powder (CP) increased, the L* and a* values tended to decrease, whereas the b*, chroma, and hue angle values tended to increase. These changes indicate that CP incorporation affected the lightness, color saturation, and hue of the samples. The decrease in L* indicates reduced lightness, while the decrease in a* toward negative values reflects a shift toward green. In contrast, the increase in b* indicates a greater contribution of yellow to the overall color. The increase in chroma suggests greater color saturation, whereas the increase in hue angle indicates a shift in hue toward the yellow-green region of the CIELAB color space. These changes may be associated with the natural pigments present in CP, particularly chlorophyll, which contributes to its green color, as well as other pigments that may contribute to the yellow component of the samples [33], as well as the higher proportion of cactus powder incorporated into the flour matrix. These findings are consistent with those reported by Hussain et al. [12], who observed that the incorporation of cactus powder into cookies decreased the L* value and significantly enhanced the green color of the final product. Overall, the addition of 5%, 10%, and 15% CP progressively increased the greenness of both CF and RF. These findings provide baseline information for the potential application of CP in flour-based food products.

### 3.2. Chemical Composition

The proximate compositions of CF and RF fortified with CP are presented in Table 2. The chemical composition of CF observed in the present study was generally consistent with previous findings. Nilusha et al. [34] reported moisture, ash, fat, protein, and total carbohydrate contents of 4.45–9.91%, 1.01–2.06%, 0.21–0.64%, 1.09–1.70%, and 89.53–90.93%, respectively, in cassava flour. Similarly, previous studies have reported total starch contents of approximately 78–82% in cassava flour [35,36]. The chemical composition of RF was also generally comparable to previously reported values. Blasé [37] reported moisture, crude protein, crude fat, crude fiber, and ash contents of 5.80–12.20%, 6.00–7.64%, 0.22–2.10%, 0.81%, and 0.14–0.81%, respectively, in rice flour. In addition, total starch contents of approximately 89–90% have been reported in previous studies [38,39]. Although the protein content of RF in the present study was slightly lower than some previously reported values, Tanimoto et al. [38] reported comparable protein contents of approximately 5.8–5.9%. Differences in proximate composition may be associated with variations in growing conditions [40]. In some rice flour formulations, total starch content exceeded the available carbohydrate content estimated by difference. Similar observations were reported by Ng and Henry [41] in certain starch samples. In the present study, available carbohydrate was calculated from proximate composition after subtracting moisture, ash, lipid, protein, and crude fiber, whereas total starch was determined using an anthrone–sulfuric acid colorimetric assay. As highlighted by McCleary et al. [42], total starch and available carbohydrates represent different analytical fractions and should be distinguished when interpreting carbohydrate composition. In addition, anthrone-based assays may be affected by non-starch carbohydrates if these compounds are not adequately removed during sample preparation. Chow and Landhäusser [43] discussed potential analytical complications associated with anthrone-based carbohydrate determination in plant tissues. These methodological considerations may contribute to differences between the reported values, although their specific effects were not evaluated in the present study. Therefore, the observed discrepancies, particularly where total starch exceeded the calculated total carbohydrate content, should be interpreted cautiously.

The incorporation of CP at 5%, 10%, and 15% increased the moisture, ash, fat, protein, and crude fiber contents of both CF and RF, whereas carbohydrate and total starch contents decreased with increasing CP concentration. In particular, crude fiber content increased from 0.32 to 3.41 g/100 g in the CF system and from 0.73 to 2.55 g/100 g in the RF system. These changes were primarily associated with the chemical composition of CP, which contained higher levels of ash, protein, and crude fiber than the unfortified flours. Previous studies have also highlighted the nutritional composition of *Opuntia ficus-indica* cladodes, particularly their fiber and mineral contents [7,33]. Santos et al. [44] reported a total dietary fiber (TDF) content of 47.70 g/100 g in freeze-dried *O. ficus-indica* cladodes, comprising 13.54 g/100 g of soluble dietary fiber (SDF) and 34.16 g/100 g of insoluble dietary fiber (IDF). Similarly, Hernández-Urbiola et al. [45] reported SDF and IDF contents of 5.50–8.84 and 40.14–58.20 g/100 g, respectively. These findings highlight the diverse fiber composition of cactus cladodes, including soluble components such as mucilage and pectin [46]. However, as only crude fiber was determined in the present study, the contributions of individual dietary fiber fractions remain to be further investigated.

The reduction in carbohydrate content may be attributed to the increased proportions of other constituents, including crude fiber, protein, ash, and fat. Similarly, the decrease in total starch content may be explained by the partial replacement of starch-rich flours with CP, resulting in a starch dilution effect. These findings are consistent with those of Silva et al. [33], who reported increased protein, mineral, and dietary fiber contents following the incorporation of cactus powder into pasta. Similarly, Nabil et al. [47] reported changes in the chemical composition of biscuits supplemented with increasing levels of *O. ficus-indica* cladode powder. The observed compositional changes may contribute to the nutritional characteristics and functional properties of the flour systems. Overall, CP incorporation modified the proximate composition of both CF and RF, particularly by increasing crude fiber content while reducing the proportions of carbohydrates and total starch.

### 3.3. Bioactive Compounds and Antioxidant Activities

Bioactive compounds are important constituents of foods due to their potential health-promoting properties, particularly their antioxidant capacity, which may help reduce oxidative damage caused by free radicals [18,48]. The bioactive compound contents and antioxidant activities of CF and RF fortified with CP are presented in Table 3. The CP used in this study contained a TPC of 386.94 mg GAE/100 g DW and a TFC of 741.85 mg QE/100 g DW. Its antioxidant activities, determined by the DPPH and FRAP assays, were 0.84 mg AA/100 g DW and 956.82 mg FeSO_4_/100 g DW, respectively. Compared with previous studies, the edible cactus powder exhibited a total phenolic content (TPC) comparable to that reported for cladodes of the same species [22]. Meanwhile, the total flavonoid content (TFC) was higher than that reported by Valenzuela-García et al. [49] for *Opuntia ficus-indica* (nopal) cladodes. This difference may be attributed to several factors, including plant cultivar, maturity stage, growing conditions, and variations in sample preparation and analytical methods.

The incorporation of CP increased (*p* < 0.05) the TPC, TFC, and antioxidant activities, as measured by the DPPH and FRAP assays, in both cassava and rice flours. These parameters increased progressively with increasing CP levels, reaching the highest values in samples containing 15% CP. In CF, TPC, TFC, DPPH, and FRAP values ranged from 77.74 to 159.35 mg GAE/100 g DW, 51.58 to 127.49 mg QE/100 g DW, 0.18 to 0.48 mg AA/100 g DW, and 423.49 to 465.84 mg FeSO_4_/100 g DW, respectively. The corresponding values for RF ranged from 119.34 to 199.61 mg GAE/100 g DW, 47.99 to 126.46 mg QE/100 g DW, 0.44 to 0.61 mg AA/100 g DW, and 481.92 to 647.41 mg FeSO_4_/100 g DW, respectively. These findings are consistent with those reported by Gutiérrez-Tlahque et al. [11], who observed that the incorporation of cactus cladodes into pasta increased the phenolic content and antioxidant capacity of the final product. Similarly, Silva et al. [33] reported that the addition of cactus to food products enhanced their bioactive properties and antioxidant activity. The increases observed in the present study may be attributed to the naturally occurring phenolic compounds, flavonoids, and other phytochemicals present in cactus cladodes, which are recognized as major contributors to antioxidant activity [10,50]. Differences between the two flour systems were also observed. RF generally exhibited higher TPC, DPPH, and FRAP values than CF at the same level of CP incorporation, whereas TFC values were comparable between the two flours. These differences may be associated with the distinct chemical compositions and structural characteristics of the flours, which could influence the retention, release, or extractability of bioactive compounds during analysis. It should be noted that the experimentally determined TPC and DPPH values of the CP-fortified flour blends were higher than those estimated from the weighted averages of their individual components. Although identical extraction conditions were applied to all samples, the measured values may be influenced by matrix-dependent differences in extraction efficiency and the responses of bioactive compounds to the analytical reagents [51]. Furthermore, sample color and turbidity may interfere with spectrophotometric measurements, particularly in the DPPH assay [52]. Therefore, these potential factors should be considered when interpreting the observed results, and further studies are warranted to better understand the trends in bioactive compound contents and antioxidant activities in CP-fortified flour systems. Overall, CP incorporation was associated with increased measured bioactive compound contents and antioxidant activity in both CF and RF systems. These findings provide preliminary insights into the bioactive characteristics of CP-fortified flour blends and their potential applications in flour-based food formulations.

### 3.4. Structural Analysis

#### 3.4.1. Microstructure

**Figure 2:**
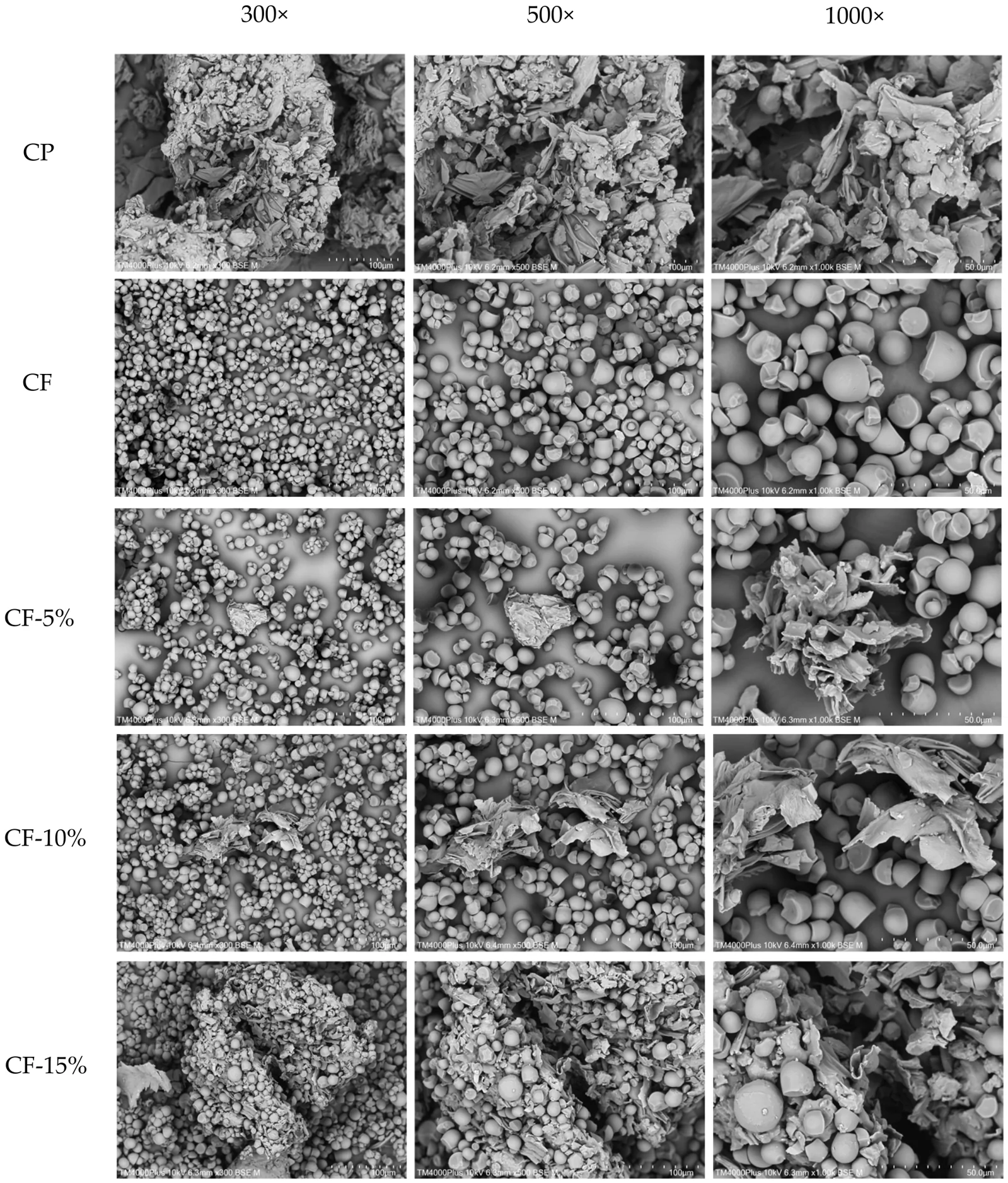
Scanning electron microscopy (SEM) images of cassava flour fortified with edible cactus powder. CP, edible cactus powder; CF, cassava flour. Furthermore, 5%, 10%, and 15% indicate the levels of edible cactus powder added to the cassava flour.

**Figure 3:**
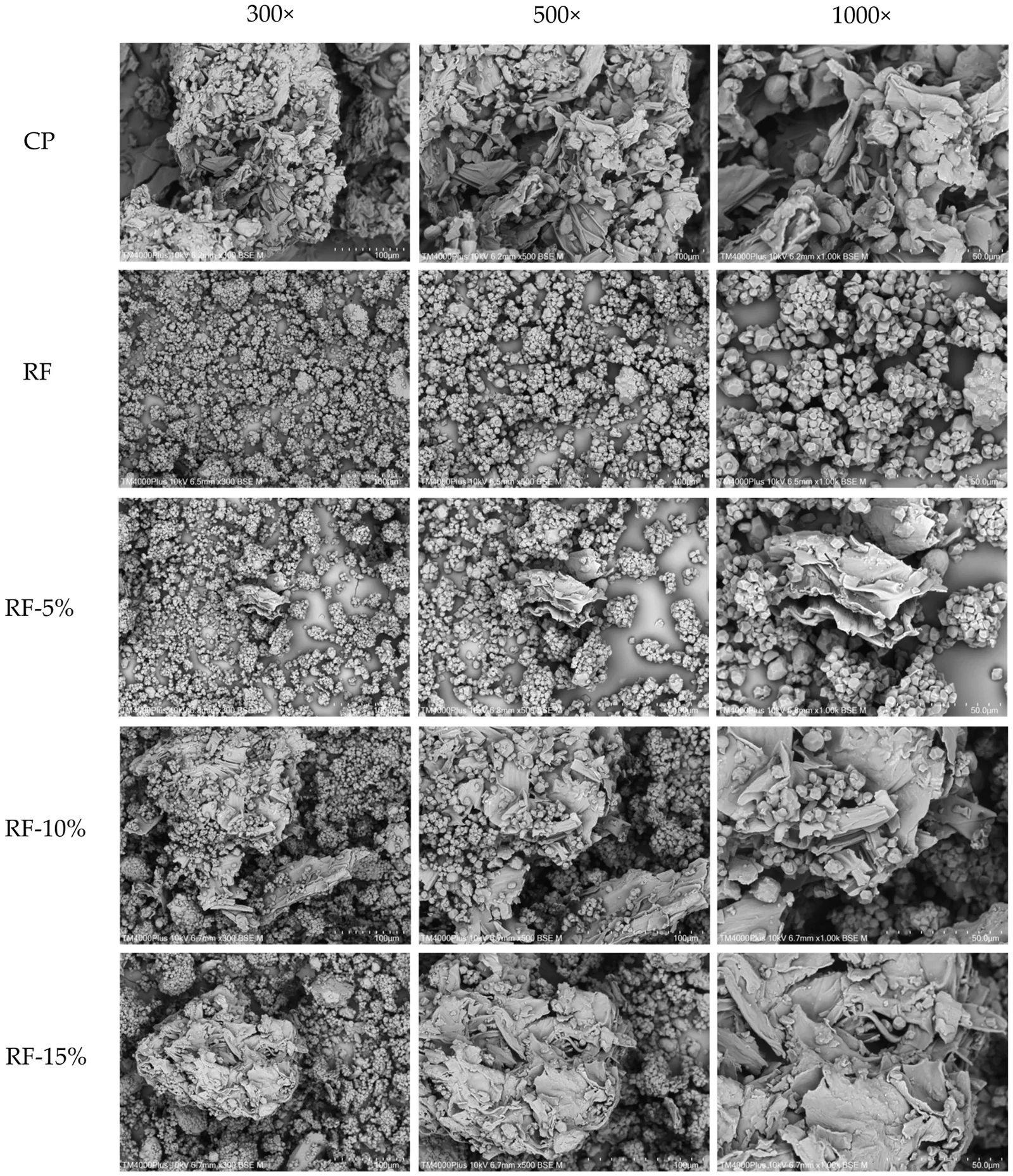
Scanning electron microscopy (SEM) images of rice flour fortified with edible cactus powder. CP, edible cactus powder; RF, rice flour. Furthermore, 5%, 10%, and 15% indicate the levels of edible cactus powder added to the rice flour.

The microstructures of CF and RF fortified with CP are presented in Figure 2 and Figure 3, respectively. The SEM observations showed that the incorporation of CP altered the morphological characteristics of both flour systems. The CP exhibited a fibrous morphology with plant cell wall fragments and porous structures, which is consistent with the microstructural characteristics reported for *Opuntia ficus-indica* by Otálora et al. [53] and *Opuntia megacantha* by Ouhammou et al. [54]. The incorporation of CP resulted in similar morphological changes in both CF and RF systems. As the CP level increased from 5% to 10% and 15%, more fibrous structures and plant cell wall materials were observed within the starch matrix, resulting in a more heterogeneous surface morphology. CF predominantly retained round to oval-shaped starch granules with relatively smooth surfaces, whereas RF exhibited polygonal-shaped granules. In both CF and RF systems, fibrous structures or CP-derived materials were observed between starch granules, together with some aggregation of starch granules and distribution of fibrous components among the granules, particularly in the samples containing 15% CP. These structural changes may be associated with the incorporation of cactus-derived components, such as fiber content and plant cell wall materials [7]. As shown in Table 2, the incorporation of increasing levels of CP into CF and RF resulted in higher crude fiber content compared with the respective control samples. The presence of these components may modify the physical organization of the flour systems, resulting in a more composite-like microstructure [55]. Similar observations were reported by Guimarães et al. [56], who found that the incorporation of fiber content can alter the structure and mechanical properties of starch-based matrices, potentially through interactions between the fiber and starch matrix. These structural characteristics, including pores associated with the incorporation of fiber content and the rough surface features of plant cell wall-derived fibrous components, may influence the functional properties of starch-based systems, particularly their flow behavior and in vitro starch digestibility [57].

Overall, the incorporation of CP modified the morphology of both cassava and rice flour systems by introducing fiber-like and cell wall structures into the flour matrix. These physical changes may influence the functional properties of the flour systems and could be relevant to the application of CP in flour-based food products.

#### 3.4.2. FT-IR Analysis

**Figure 4:**
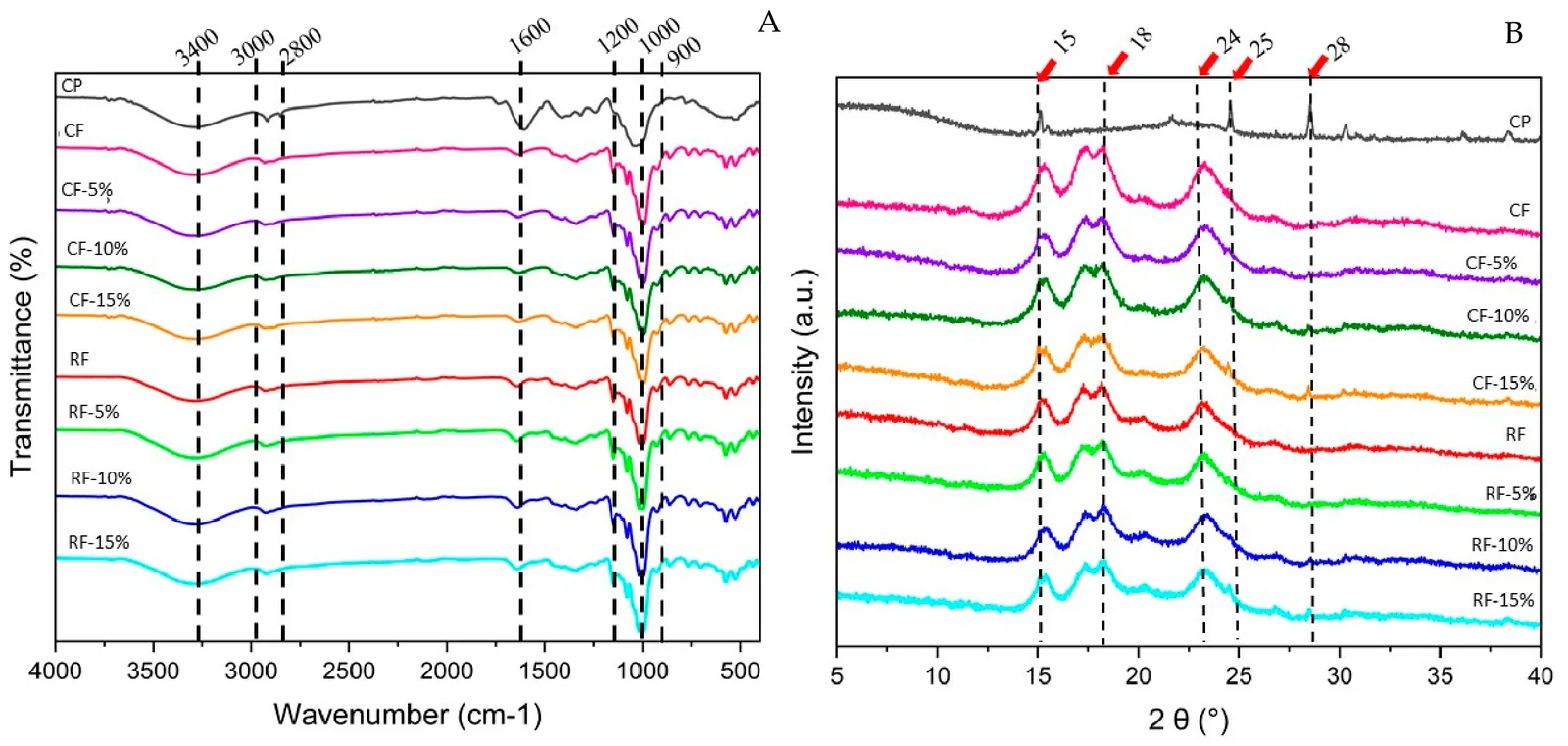
Fourier−transform infrared (FT−IR) spectra (**A**) and X−ray diffraction (XRD) patterns (**B**) of cassava flour and rice flour fortified with edible cactus powder. CP: edible cactus powder; CF: cassava flour; and RF: rice flour. Similarly, 5%, 10%, and 15% indicate the levels of edible cactus powder added to the cassava flour and rice flour.

Figure 4A shows the FTIR spectra of CF and RF fortified with CP. All samples exhibited absorption bands over the range of 400–4000 cm^−1^, reflecting the characteristic functional groups of starch and other polysaccharide components present in CP. The CP sample exhibited a broad absorption band at 3200–3600 cm^−1^, which was associated with O–H stretching vibrations and hydrogen-bonded hydroxyl groups in carbohydrate-rich components. The absorption band at 2840–3000 cm^−1^ was attributed to C–H stretching vibrations associated with cellulose and hemicellulose [58]. In addition, an absorption band at approximately 1600–1640 cm^−1^ was observed, which may be associated with vibrations of carboxyl- and ester-containing components present in cellulose and other polysaccharides, including pectin. These spectral characteristics are consistent with those reported for *Opuntia ficus-indica* by De Farias et al. [59]. The bands observed at 1350–1400 cm^−1^ were assigned to vibrations associated with carboxylate (COO^−^) groups, which are commonly present in plant cell-wall components such as cellulose and hemicellulose [22]. Furthermore, the bands at 850–900 cm^−1^ may be related to α-glycosidic linkages commonly present in plant polysaccharides, including pectin [58,60]. When comparing the two native starches, CF exhibited a relatively prominent absorption band at 1022 cm^−1^, which has been associated with C–O stretching vibrations in the amorphous regions of starch [61]. In comparison with CF, RF exhibited relatively stronger absorption bands in the regions of 3200–3600 and approximately 1640 cm^−1^. These differences may be associated with differences in moisture content and the local molecular environment of hydroxyl groups and water molecules. Such absorption bands are commonly observed in carbohydrate-rich materials [62,63]. This observation was consistent with the chemical composition data presented in Table 2, which showed a higher moisture content in RF than in CF.

The addition of CP altered the FTIR absorption profiles of both the CF and RF systems. Increasing the CP concentration to 5%, 10%, and 15% resulted in progressively greater absorption intensity in the regions of 3200–3600, approximately 2900, and 1600 cm^−1^. These regions were mainly associated with O–H stretching, C–H stretching, and O–H bending of absorbed water and/or C=O-related vibrations, respectively. The corresponding absorption features may originate from water and non-starch components present in CP, including cellulose and pectin [22,58,63]. The increased strength of these bands may be related to the greater contribution of CP components to the starch. As shown in Table 2, increasing the CP concentration increased the contribution of moisture and fiber to both CF and RF systems, which may have contributed to the enhanced absorption intensity in these regions. A similar observation was reported by Rivera-Corona et al. [64], who found that the incorporation of cactus mucilage into sorghum flour affected the intensity of the absorption band at approximately 3308 cm^−1^. This band was associated with O–H vibrations originating from starch, cactus mucilage, and water. The spectral characteristics also appeared to vary with the concentration of CP incorporated into the starch systems. A similar observation was reported by Zhu et al. [57], report that the incorporation of cactus-derived polysaccharides into rice starch at concentrations of 0.6–3% (*w*/*w*) did not result in the appearance of new characteristic bands, which indicated no detectable formation of new chemical functionalities between cactus polysaccharides and rice starch.

Overall, CP incorporation into CF and RF modified the intensity of characteristic FTIR absorption bands, particularly in regions associated with hydroxyl-, water-, and carbohydrate-related vibrations. However, no new characteristic absorption bands or substantial shifts in the major characteristic peaks were observed. Thus, the FTIR results indicate changes in the spectral characteristics of the starch–CP physical mixtures without evidence of a new chemical structure. These findings provide preliminary information on the incorporation of CP into cassava and rice flour systems.

#### 3.4.3. X-Ray Diffraction Patterns

X-ray diffraction (XRD) analysis was performed to characterize the diffraction patterns of CF and RF fortified with CP. The XRD patterns are presented in Figure 4B. Both CF and RF exhibited characteristic diffraction peaks at approximately 2θ = 15.11°, 17.09°, 17.92°, and 23.00°, which are commonly observed in starch-based materials and have been reported for cassava and rice starch systems [62,65]. Comparison between the two flour types showed that the diffraction peaks of CF around 15° and 17° were broader than those of RF, indicating differences in the diffraction profiles of the two starch materials [66]. These differences are presented here in terms of their diffraction characteristics rather than as evidence of differences in crystalline structure. The CP sample exhibited diffraction peaks at approximately 15°, 22°, 25°, and 28°, which were generally consistent with previously reported XRD patterns of cactus materials from the same genus [22]. The diffraction features around 14–15° and 22° have been reported in association with cellulose I-containing plant materials, whereas the diffraction features observed around 24–25° and 28° may be related to other components present in cactus tissues, including plant-fiber-associated and inorganic components [67].

Incorporation of CP at concentrations of 5%, 10%, and 15% into both CF and RF resulted in more pronounced diffraction features in the regions around 2θ = 24–25° and 28–30°, with increasing intensity as the CP concentration increased. These changes may reflect the contribution of cactus-derived components, including dietary fiber and inorganic constituents, to the overall diffraction patterns of the flour–CP mixtures. Similar diffraction features have been reported in cactus-containing materials and have been associated with cellulose-related components and calcium-containing mineral phases [22,59]. These findings are consistent with those reported by De Farias et al. [59] who reported that increasing cactus powder incorporation in films enhanced the diffraction features around 2θ = 28°, which were potentially associated with mineral crystalline phases, including calcium oxalate monohydrate. The relatively high ash content of CP observed in the present study may also indicate the contribution of inorganic constituents to its diffraction pattern. Calcium oxalate is of particular nutritional interest because it is recognized as an antinutritional compound. Missaoui et al. [8] reported the presence of calcium (75.18 mg/g), sodium (19.18 mg/g), potassium (16.84 mg/g), magnesium (13.80 mg/g), and oxalate (1.05 mg/g) in *Opuntia ficus-indica* cladodes. Oxalates may reduce calcium bioavailability by forming poorly soluble complexes; however, their nutritional significance depends on their concentration and dietary exposure. Furthermore, Hernández-Becerra et al. [47] discussed previous findings of oxalate-to-calcium molar ratios below 1 across different maturity stages of *O. ficus-indica* cladodes, suggesting that the potential effects on calcium availability may depend on the relative concentrations of these compounds. Nevertheless, calcium oxalate was not directly confirmed or quantified in the present study, and its potential antinutritional implications in CP require further investigation.

The observed diffraction patterns were also consistent with the chemical composition presented in Table 2. Increasing CP incorporation increased the ash and crude fiber content of the flour systems, which may explain the greater contribution of diffraction features associated with cactus-derived fiber and inorganic components [22]. Nevertheless, the major diffraction pattern of the starch component remained generally similar after CP incorporation, although variations in the intensity of several diffraction peaks were observed, particularly in the regions around 2θ = 24–25° and 28–30°. These changes became more apparent with increasing CP concentration. A similar observation was reported by Zhu et al. [57], who found that the incorporation of cactus powder into rice starch altered the intensity of diffraction peaks while the characteristic diffraction pattern of the starch system remained generally unchanged after mixing; they also observed no clearly distinguishable new diffraction peaks attributable to cactus powder incorporation.

Overall, CP incorporation altered the diffraction profiles of both cassava and rice flour systems, reflecting the contribution of cactus-derived components, particularly fiber content and inorganic constituents. These changes indicate alterations in the overall diffraction characteristics of the physical starch–CP mixtures rather than the formation of new crystalline or chemical structures.

### 3.5. Pasting Properties

Pasting properties are important characteristics in the processing of starch-based food products. The pasting properties of CF and RF fortified with CP are presented in Table 4. The incorporation of CP at levels of 5%, 10%, and 15% influenced the peak viscosity (PV), trough viscosity (TV), breakdown (BD), final viscosity (FV), setback (SB), and pasting temperature (PT) of both flour systems.

Increasing CP levels generally decreased PV, TV, BD, and FV, while slightly increasing PT. PV decreased by approximately 20% and 23% in CF and RF, respectively, while TV decreased by 21% and 25%. BD decreased by approximately 20% in CF and 15% in RF, whereas FV decreased by 19% and 24%, respectively. PT increased from 77.87 to 80.20 °C in CF and from 85.12 to 89.70 °C in RF. In contrast, SB exhibited a nonmonotonic response in the CF system but generally decreased in the RF system, indicating that the effect of CP on starch reassociation during cooling differed between the two flour matrices. These findings are consistent with those of Klinmalai et al. [68], who reported that the incorporation of polysaccharides from mung bean peel into rice starch reduced PV and BD. These reductions may be related to restricted starch swelling and water absorption, which can affect the pasting behavior of the rice starch system. This effect may be associated with non-starch components, particularly fiber content. Similarly, Zhu et al. [57] reported that the incorporation of cactus-derived polysaccharides reduced the PV and FV of rice starch. The reductions in PV, TV, BD, and FV with increasing CP levels may indicate restricted starch swelling and altered starch–water interactions in the composite systems [68]. Because the RVA measurements were performed using the same mass of each flour blend, partial replacement of CF or RF with CP reduced the amount of starch present in the test suspension. This starch dilution effect may have contributed to the lower pasting viscosities. In addition, CP-derived non-starch components may influence water distribution and starch swelling during heating [5,55]. As shown in Table 2, increasing CP incorporation decreased the carbohydrate content calculated by difference and total starch content while increasing crude fiber content. Therefore, the observed changes in pasting properties may reflect the combined contributions of starch dilution and the effects of CP-derived components, although their individual contributions could not be distinguished in the present study. The lower pasting viscosities were also consistent with the reduced apparent viscosity observed in the rheological measurements. This interpretation is consistent with Gan et al. [69], who reported that fibers can compete with starch granules for water during heating, thereby restricting starch swelling and reducing PV. The lower BD may reflect a smaller reduction in viscosity during the holding phase, which may be associated with limited starch swelling in the presence of CP. Meanwhile, fiber content may affect the reassociation of starch chains during cooling, which may contribute to the lower FV observed in the CP-supplemented systems.

Overall, CP incorporation altered the pasting behavior of both CF and RF, potentially through the combined contributions of starch dilution and changes in water distribution associated with CP-derived non-starch components.

### 3.6. Rheological Properties

#### 3.6.1. Frequency Sweep Tests

**Figure 5:**
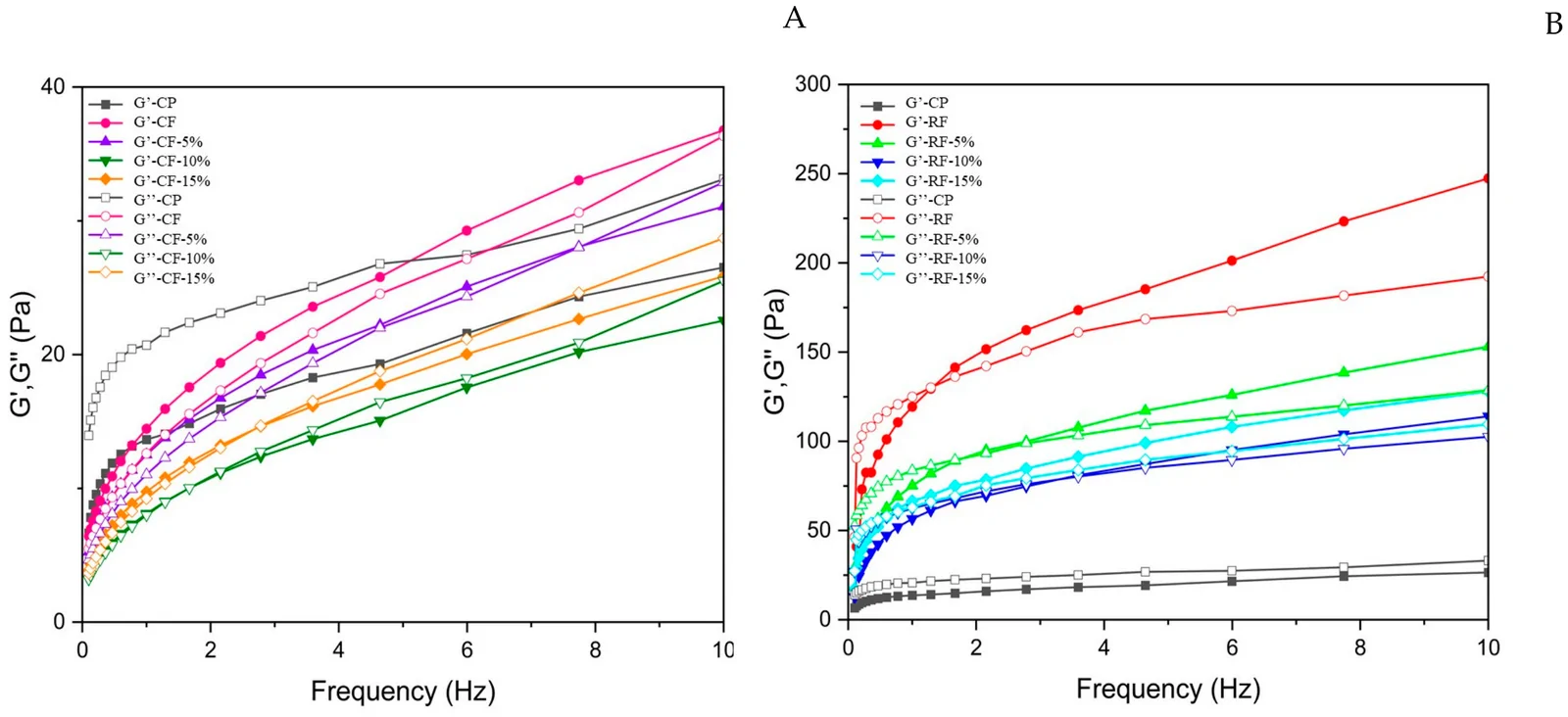
Dynamic rheological properties, including storage modulus (G′) and loss modulus (G″), of (**A**) cassava flour (CF) and (**B**) rice flour (RF) fortified with edible cactus powder (CP). CP, edible cactus powder; CF, cassava flour; RF, rice flour. The concentrations of 5%, 10%, and 15% indicate the levels of CP added to CF or RF.

The frequency sweep results of CF and RF fortified with CP are presented in Figure 5. Both the storage modulus (G′) and loss modulus (G″) generally increased with increasing frequency, indicating frequency-dependent viscoelastic behavior. CP incorporation generally reduced G′ and G″ compared with the respective flour controls. However, the relationships between the two moduli differed between the flour systems and varied with CP concentration and frequency. In the CF system, the relative magnitudes of G′ and G″ varied with frequency, particularly in the control and 5% CP formulations. At higher CP concentrations (10% and 15%), G″ generally exceeded G′ over most of the investigated frequency range, suggesting predominantly viscous behavior under these conditions. In the RF system, G″ exceeded G′ at lower frequencies, while the separation between the moduli changed with increasing frequency. Crossovers were observed in some formulations, with G′ exceeding G″ at higher frequencies, whereas other formulations showed more comparable moduli. Thus, the RF formulations exhibited frequency-dependent viscoelastic responses rather than uniformly elastic behavior throughout the investigated range. The differences between CF and RF may be associated with variations in starch composition and physicochemical characteristics, which can influence swelling, gelatinization, and the development of viscoelastic structures [61,70,71]. The overall reduction in G′ and G″ following CP incorporation indicates lower viscoelastic moduli of the gelatinized flour pastes. Similar findings were reported by Zhu et al. [57], who observed a reduction in the elastic modulus of rice starch supplemented with Opuntia dillenii powder. These changes may be associated with the partial replacement of starch-rich flour by CP and the presence of non-starch components, including dietary fiber and mucilaginous polysaccharides, which may influence water distribution and the organization of components within the gelatinized flour systems [72]. Nevertheless, the relative contributions of starch dilution and specific interactions between CP constituents and starch could not be distinguished in the present study.

CP alone exhibited G″ > G′ throughout the investigated frequency range, indicating a predominantly viscous response under the measurement conditions. This behavior is consistent with reports on certain plant-derived polysaccharide systems, in which viscous contributions exceed elastic contributions depending on their composition and structural characteristics [73]. Similarly, Morales-Martínez et al. [74] reported that pectin extracted from Opuntia exhibited G″ > G′, suggesting predominantly viscous behavior. Furthermore, Song et al. [75] reported that non-starch components, including proteins, lipids, and dietary fiber, can influence the rheological properties of starch-based systems. Therefore, the rheological effects of CP may depend on both the flour matrix and the composition of the incorporated material.

Overall, CP incorporation modified the frequency-dependent viscoelastic behavior of the gelatinized CF and RF pastes. The CF formulations containing 10% and 15% CP exhibited predominantly viscous behavior over most of the investigated frequency range, whereas the RF formulations showed frequency-dependent changes in the relative contributions of elastic and viscous responses. These findings may inform further formulation studies involving CP-fortified gluten-free food systems with specific viscoelastic requirements.

#### 3.6.2. Flow Behavior

**Figure 6:**
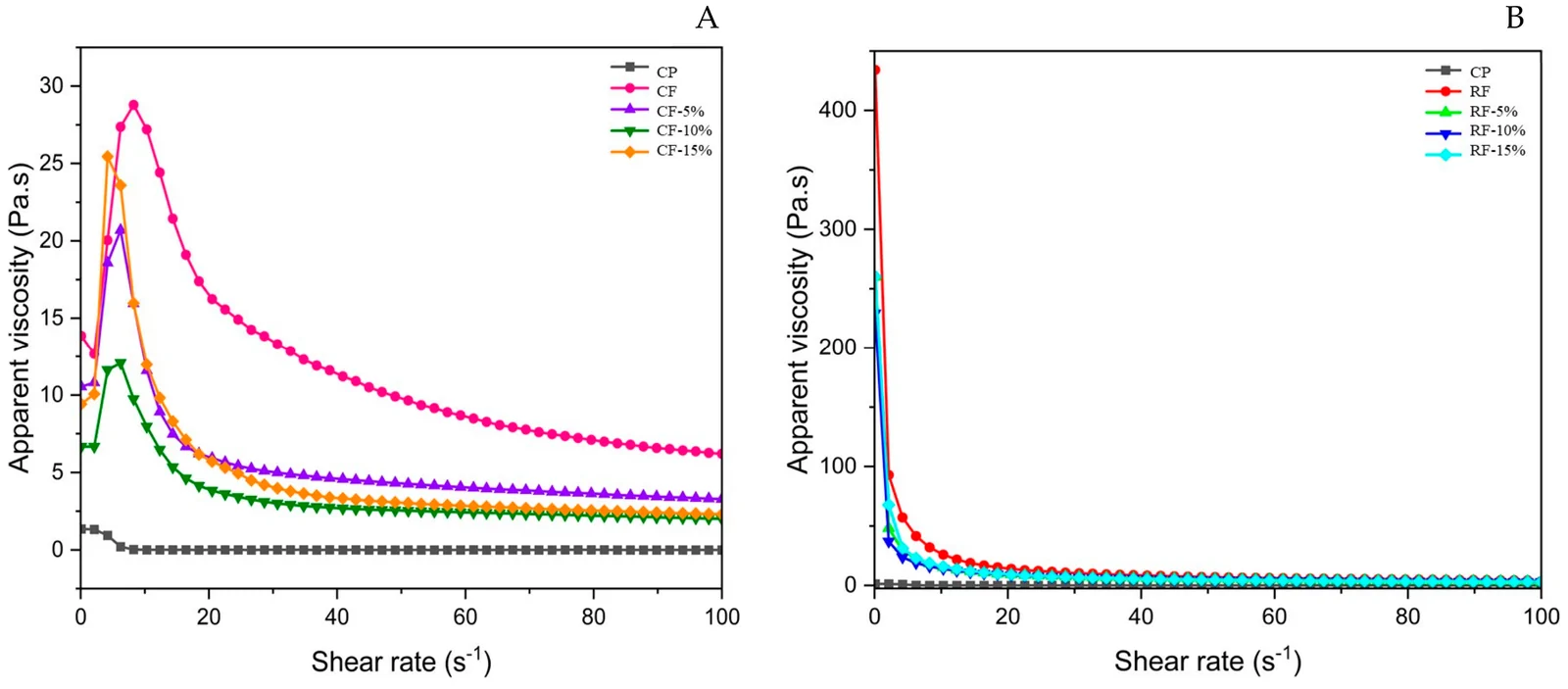
Apparent viscosity of (**A**) cassava flour (CF) and (**B**) rice flour (RF) fortified with edible cactus powder (CP). CP, edible cactus powder; CF, cassava flour; RF, rice flour. The concentrations of 5%, 10%, and 15% indicate the levels of CP added to CF or RF.

Flow behavior is an important rheological property relevant to food processing and is commonly characterized by apparent viscosity as a function of shear rate. The shear-dependent flow behavior of CF and RF fortified with CP is presented in Figure 6. The incorporation of CP affected the flow behavior of both flour systems. As the shear rate increased, the apparent viscosity of the samples decreased, indicating non-Newtonian shear-thinning behavior. Increasing the CP concentration to 5%, 10%, and 15% resulted in a decrease in apparent viscosity in both CF and RF across the tested shear-rate range. This finding is consistent with Zhu et al. [57], who reported that the incorporation of *Opuntia dillenii* powder into rice starch reduced the apparent viscosity while maintaining non-Newtonian shear-thinning behavior. The decrease in apparent viscosity following CP incorporation indicates reduced flow resistance under the applied shear conditions. The decrease in apparent viscosity may partly reflect starch dilution, as the pastes were prepared using the same mass of each flour blend, and CP replacement reduced the starch content of the systems. In addition, non-starch components in CP, including fiber and cell-wall polysaccharides such as pectic substances and mucilage [9], which may modify water distribution and component interactions within the flour matrix [58]. As shown in Table 2, CP incorporation increased crude fiber content while decreasing total starch content. Therefore, the observed changes in apparent viscosity may reflect the combined contributions of starch dilution and CP-derived components, although their individual contributions could not be distinguished under the present experimental conditions [76]. The shear-dependent flow results were also consistent with the dynamic rheological properties shown in Figure 5. The decrease in apparent viscosity following CP incorporation was accompanied by decreases in both G′ and G″, suggesting that CP addition was associated with reduced flow resistance and lower viscoelastic moduli of the flour systems. Thus, CP incorporation affected both the shear-dependent flow behavior and viscoelastic response of the systems. When comparing the two flour types, CF exhibited lower apparent viscosity than RF across the tested shear-rate range. This difference may be related to the intrinsic structural characteristics of the starches, particularly starch granule characteristics and morphology [77]. Differences in starch granule structure may be one of the factors contributing to the different rheological behaviors of CF and RF [5,71].

Overall, CP incorporation reduced the apparent viscosity of both CF and RF while maintaining shear-thinning behavior, with CF showing lower apparent viscosity than RF across the tested shear-rate range. These flow characteristics may be relevant to the formulation and processing of gluten-free food systems where controlled flow behavior is desirable.

### 3.7. Freeze–Thaw Stability

Freeze–thaw stability was assessed to evaluate gel stability and water retention during freezing and thawing [57]. The freeze–thaw stability results of CF and RF gels fortified with CP are presented in Table 5. The results showed that the level of CP incorporation affected gel syneresis in both CF and RF systems. Compared with the control samples, the incorporation of CP at 5%, 10%, and 15% reduced syneresis in both CF and RF gels, with the 15% CP treatment showing the greatest reduction. This reduction in syneresis may be associated with the water-absorbing and water-retaining components of CP, particularly fiber content and hydrocolloid-like polysaccharides such as pectin, which may enhance water retention and reduce water mobility within the gel structure [78]. Interestingly, the CP sample exhibited syneresis values of 45.02–66.43%, which were higher than those of the CF control but lower than those of the RF control at the corresponding freeze–thaw cycles. However, CP incorporation reduced syneresis relative to the respective controls in both flour systems. These results suggest that the effect of CP on water retention may depend on the flour matrix and the combined contributions of starch and CP-derived components.

The incorporation of CP into the starch matrix may alter water distribution and mobility through interactions between starch and fiber content or pectic polysaccharides [57]. In addition, the structural characteristics of fiber and plant cell-wall polysaccharides may influence water retention and network development within the matrix [9,55]. These findings are consistent with the study by Zhu et al. [57], which reported that the incorporation of cactus powder from *Opuntia dillenii* into rice starch gels reduced syneresis following freeze–thaw treatment. This finding further suggests that water-retaining components in cactus powder may contribute to maintaining water within the gel structure.

However, syneresis tended to increase with an increasing number of freeze–thaw cycles. This increase may be associated with structural changes and molecular rearrangement of starch during repeated freezing and thawing, together with the formation and growth of ice crystals. These processes may disrupt the gel structure and promote water migration from the gel matrix, resulting in increased syneresis [68]. The syneresis values of the CF and RF samples ranged from 26.33 to 44.96% and 65.58–76.79%, respectively. The RF gels exhibited greater syneresis than the CF gels, which may be related to differences in starch structure and molecular organization between the two starch sources, resulting in differences in water retention and responses to freeze–thaw treatment [5,26].

Overall, the incorporation of CP into CF and RF systems may improve water retention and gel stability during freeze–thaw treatment. These findings suggest that CP-fortified starch systems may have potential for applications in food products that undergo freezing and thawing processes.

### 3.8. In Vitro Starch Digestibility

The changes in vitro starch digestibility of cassava flour (CF) and rice flour (RF) supplemented with cactus powder (CP) are presented in Table 6. All flour samples were analyzed in their native, uncooked state without prior thermal treatment. Compared with the respective controls, increasing CP incorporation from 5% to 15% resulted in a progressive reduction in the proportion of rapidly digestible starch (RDS), accompanied by an increase in slowly digestible starch (SDS) in both CF and RF systems. CP incorporation also affected the proportion of resistant starch (RS), although different trends were observed between the two flour systems. In CF, RS increased from 36.25% in the control to 51.08% at 5% CP incorporation and remained relatively stable at 48.82% and 48.74% with 10% and 15% CP incorporation, respectively. In contrast, RS in RF increased from 27.37% in the control to 34.83–36.34% following CP incorporation. These findings suggest a shift in the measured starch digestibility fractions toward lower RDS and higher SDS and RS proportions, consistent with the findings of Zhu et al. [57], who reported increased RS and SDS proportions following the incorporation of Opuntia dillenii powder. The observed changes in starch digestibility may be associated with the polysaccharide components of CP, including mucilage, pectin, and hemicellulose [9], which could modify the starch matrix and influence starch accessibility to digestive enzymes [55]. When incorporated into starch-rich systems, these components may contribute to physical barriers that restrict enzymatic access to starch granules [57]. This proposed mechanism is consistent with the increased crude fiber content presented in Table 2 and the SEM observations in Figure 2 and Figure 3, which show CP particles distributed among and partially covering the starch granules. Nevertheless, these observations provide only indirect evidence of potential fiber–starch interactions. The partial replacement of CF and RF with CP may also contribute to a starch dilution effect. Although the digestibility fractions were normalized to the total starch content of each formulation, the potential contributions of starch dilution and fiber–starch interactions cannot be fully distinguished.

However, it is important to note that in this study, the relatively high RS values observed should be interpreted with caution. The flour blends were analyzed in their uncooked state without prior thermal treatment or gelatinization, which may have limited enzyme accessibility due to the structural characteristics of native starch granules. Previous studies have reported RS values ranging from 43% to 60% in uncooked flour samples [79]. Similarly, Seephua et al. [31] reported an RS value of 42.12% in uncooked rice noodles, which is comparable to the values observed in the present study. Previous studies have also reported relatively high RS values in uncooked flour samples. Nevertheless, RS was calculated by difference and may therefore include starch that was not completely hydrolyzed under the experimental conditions. Incomplete starch hydrolysis may be influenced by several factors, including the structural characteristics of native starch granules and the efficiency of enzymatic digestion. Furthermore, the DNS method measures reducing sugars, including glucose, maltose, and reducing oligosaccharides, rather than glucose specifically. Differences in the analytical responses of individual reducing sugars may therefore introduce uncertainty into the estimated starch digestibility fractions. In addition, CP incorporation introduces non-starch components that may contribute to the measured reducing sugar content and potentially influence the estimated starch digestibility fractions. Although the initial reducing sugar content was accounted for in the calculations, potential interference from CP-derived components during enzymatic digestion cannot be completely excluded.

Moreover, the absence of a cooked-sample comparison represents a limitation of the present study, as thermal processing can substantially influence starch digestibility. Therefore, the findings should be interpreted within the context of uncooked flour systems and should not be directly extrapolated to thermally processed food products. Future studies involving cooked samples and glucose-specific analytical methods could provide a more comprehensive understanding of starch digestion behavior under different processing conditions. Overall, CP-fortified flour blends exhibited changes in the estimated starch digestibility fractions, with generally lower RDS and higher SDS values, while RS showed varying responses between the CF and RF systems. These findings provide preliminary information on the starch digestibility characteristics of uncooked CP-fortified flour blends and may serve as a basis for future investigations.

## 4. Conclusions

The incorporation of CP into CF and RF modified their chemical composition, structural characteristics, pasting and rheological properties, freeze–thaw stability, and in vitro starch digestibility. CP incorporation increased crude fiber content and altered the levels of other chemical constituents and bioactive compounds in both flour systems. SEM observations revealed the presence and distribution of CP particles within the flour matrices, while FTIR and XRD analyses indicated changes in their spectral and diffraction characteristics. CP generally reduced the viscoelastic moduli (G′ and G″), apparent viscosity, and most pasting viscosity parameters, suggesting modifications in the structural and flow behavior of the flour systems. In contrast, CP incorporation reduced freeze–thaw syneresis, indicating improved stability under the experimental conditions. Furthermore, CP-fortified flour blends generally exhibited lower rapidly digestible starch (RDS) and higher slowly digestible starch (SDS) fractions, whereas resistant starch (RS) showed varying responses depending on the flour system. Nevertheless, these findings should be interpreted with consideration of the limitations associated with the in vitro starch digestibility assessment. The present study provides preliminary information on the physicochemical and functional characteristics of CP-fortified flour blends, which may serve as a basis for further food product development. Future studies should investigate the soluble and insoluble dietary fiber fractions, evaluate starch digestibility under different processing conditions, and assess the technological properties, nutritional composition, and sensory characteristics of developed food products. Overall, the findings suggest that CP incorporation may be a promising approach for modifying the functional properties of CF- and RF-based flour systems, providing a foundation for further investigation of their potential applications in gluten-free food formulations.

## Figures and Tables

**Table 1:** Color parameters of cassava flour and rice flour fortified with edible cactus powder.

Sample	L*	a*	b*	Chroma	Hue Angle
CP	64.66 ± 0.06 ^h^	−7.83 ± 0.03 ^g^	21.16 ± 0.02 ^a^	22.56 ± 0.03 ^a^	110.31 ± 0.03 ^a^
CF	93.82 ± 0.02 ^b^	−0.24 ± 0.02 ^a^	2.27 ± 0.15 ^h^	2.27 ± 0.15 ^h^	95.99 ± 0.92 ^e^
CF-5%	91.33 ± 0.01 ^c^	−2.92 ± 0.02 ^c^	8.14 ± 0.03 ^e^	8.64 ± 0.03 ^e^	109.74 ± 0.11 ^b^
CF-10%	87.51 ± 0.08 ^f^	−3.93 ± 0.02 ^f^	10.59 ± 0.04 ^c^	11.29 ± 0.04 ^c^	110.38 ± 0.06 ^a^
CF-15%	86.41 ± 0.03 ^g^	−3.96 ± 0.02 ^f^	10.56 ± 0.08 ^c^	11.28 ± 0.08 ^c^	110.38 ± 0.06 ^a^
RF	94.06 ± 0.01 ^a^	−0.23 ± 0.02 ^a^	3.04 ± 0.02 ^g^	3.05 ± 0.01 ^g^	94.38 ± 0.30 ^f^
RF-5%	90.97 ± 0.01 ^d^	−2.19 ± 0.01 ^b^	7.52 ± 0.04 ^f^	7.83 ± 0.04 ^f^	106.24 ± 0.01 ^d^
RF-10%	89.2 ± 0.23 ^e^	−3.18 ± 0.01 ^d^	9.99 ± 0.07 ^d^	10.48 ± 0.07 ^d^	107.65 ± 0.06 ^c^
RF-15%	87.5 ± 0.09 ^f^	−3.69 ± 0.04 ^e^	11.38 ± 0.09 ^b^	11.96 ± 0.09 ^b^	107.99 ± 0.07 ^c^

The values are presented as mean ± standard deviation (*n* = 3). Superscripts ^a–h^ indicate that the means in the same column are significantly different (*p* < 0.05). CP, edible cactus powder; CF, cassava flour; RF, rice flour. Furthermore, 5%, 10%, and 15% indicate the levels of edible cactus powder added to the cassava flour or rice flour.

**Table 2:** Proximate composition of cassava flour and rice flour fortified with edible cactus powder (g/100 g sample).

Sample	Moisture	Ash	Lipid	Protein	Crude Fiber Content	Carbohydrate	Total Starch Content
CP	11.45 ± 0.10 ^a^	15.84 ± 0.18 ^a^	0.86 ± 0.02 ^c^	8.45 ± 0.16 ^a^	17.42 ± 0.36 ^a^	45.97 ± 0.61 ^h^	53.05 ± 0.99 ^h^
CF	4.90 ± 0.23 ^f^	1.65 ± 0.09 ^f^	0.19 ± 0.01 ^g^	1.16 ± 0.03 ^g^	0.32 ± 0.01 ^g^	91.76 ± 0.27 ^a^	84.79 ± 0.23 ^c^
CF-5%	5.66 ± 0.30 ^de^	2.22 ± 0.14 ^e^	0.22 ± 0.02 ^f^	1.66 ± 0.07 ^f^	1.64 ± 0.14 ^e^	88.58 ± 0.40 ^b^	79.77 ± 0.18 ^e^
CF-10%	6.37 ± 0.27 ^c^	2.51 ± 0.16 ^d^	0.24 ± 0.01 ^ef^	1.86 ± 0.08 ^f^	2.64 ± 0.19 ^c^	86.35 ± 0.43 ^c^	76.21 ± 0.29 ^f^
CF-15%	7.49 ± 0.20 ^b^	3.03 ± 0.24 ^c^	0.28 ± 0.01 ^d^	2.29 ± 0.12 ^e^	3.41 ± 0.12 ^b^	83.48 ± 0.14 ^e^	73.70 ± 0.34 ^g^
RF	5.69 ± 0.17 ^e^	0.87 ± 0.04 ^g^	1.01 ± 0.03 ^b^	5.37 ± 0.13 ^d^	0.73 ± 0.02 ^f^	86.55 ± 0.06 ^c^	88.41 ± 0.41 ^a^
RF-5%	5.98 ± 0.25 ^d^	1.10 ± 0.06 ^g^	1.03 ± 0.01 ^b^	5.48 ± 0.18 ^d^	1.42 ± 0.09 ^e^	84.93 ± 0.25 ^d^	86.83 ± 0.19 ^b^
RF-10%	6.55 ± 0.15 ^c^	1.70 ± 0.07 ^f^	1.04 ± 0.02 ^b^	5.82 ± 0.02 ^c^	2.10 ± 0.11 ^d^	82.32 ± 0.08 ^f^	84.99 ± 0.39 ^c^
RF-15%	7.26 ± 0.06 ^b^	3.21 ± 0.30 ^b^	1.08 ± 0.03 ^a^	6.22 ± 0.18 ^b^	2.55 ± 0.18 ^c^	78.39 ± 0.59 ^g^	82.26 ± 0.26 ^d^

Values are presented as mean ± standard deviation (*n* = 3). Superscripts ^a–h^ indicate that the means in the same column are significantly different (*p* < 0.05). All compositional values are expressed as g/100 g sample on an as-is basis. Carbohydrate was calculated by difference. CP, edible cactus powder; CF, cassava flour; RF, rice flour. The values 5%, 10%, and 15% indicate the levels of CP incorporated into CF or RF.

**Table 3:** Bioactive compounds and antioxidant activities of cassava flour and rice flour fortified with edible cactus powder.

Sample	TPC (mg GAE/100 g DW)	TFC (mg QE/100 g DW)	DPPH (mg AA/100 g DW)	FRAP (mg FeSO_4_/100 g DW)
CP	386.94 ± 0.61 ^a^	741.85 ± 4.07 ^a^	0.84 ± 0.03 ^a^	956.82 ± 7.34 ^a^
CF	42.01 ± 1.01 ^g^	1.33 ± 0.08 ^f^	0.11 ± 0.01 ^h^	339.56 ± 2.90 ^i^
CF-5%	77.74 ± 1.40 ^f^	51.58 ± 2.35 ^d^	0.18 ± 0.01 ^g^	423.49 ± 4.45 ^g^
CF-10%	109.35 ± 1.22 ^e^	70.05 ± 0.89 ^c^	0.39 ± 0.01 ^f^	438.78 ± 4.13 ^f^
CF-15%	159.35 ± 0.61 ^c^	127.49 ± 4.95 ^b^	0.48 ± 0.01 ^d^	465.84 ± 5.50 ^e^
RF	38.013 ± 1.15 ^h^	17.74 ± 0.94 ^e^	0.19 ± 0.01 ^g^	390.94 ± 3.11 ^h^
RF-5%	119.34 ± 1.29 ^d^	47.99 ± 5.54 ^d^	0.44 ± 0.01 ^e^	481.92 ± 4.45 ^d^
RF-10%	161.21 ± 1.66 ^c^	73.64 ± 2.35 ^c^	0.51 ± 0.02 ^c^	578.39 ± 5.50 ^c^
RF-15%	199.61 ± 6.12 ^b^	126.46 ± 4.07 ^b^	0.61 ± 0.01 ^b^	647.41 ± 4.24 ^b^

The values are presented as mean ± standard deviation (*n* = 3). Superscripts ^a–i^ indicate that the means in the same column are significantly different (*p* < 0.05). CP, edible cactus powder; CF, cassava flour; RF, rice flour. Furthermore, 5%, 10%, and 15% indicate the levels of edible cactus powder added to the cassava flour or rice flour. DW, dry weight.

**Table 4:** Pasting properties of cassava flour and rice flour fortified with edible cactus powder.

Sample	Peak Viscosity (cp)	Trough Viscosity (cp)	Breakdown (cp)	Final Viscosity (cp)	Setback (cp)	Pasting Temperature (°C)
CP	2320.00 ± 52.68 ^i^	1447.33 ± 2.08 ^g^	872.67 ± 51.08 ^g^	1670.00 ± 2.00 ^h^	222.67 ± 3.21 ^e^	51.98 ± 0.04 ^i^
CF	5324.00 ± 40.84 ^a^	2735.00 ± 85.58 ^b^	2589.00 ± 60.23 ^a^	3622.00 ± 107.92 ^d^	887.00 ± 36.86 ^cd^	77.87 ± 0.02 ^h^
CF-5%	4889.33 ± 29.28 ^b^	2429.00 ± 35.37 ^e^	2460.33 ± 35.02 ^b^	3354.67 ± 46.36 ^e^	925.67 ± 80.52 ^c^	78.61 ± 0.16 ^g^
CF-10%	4580.00 ± 34.59 ^c^	2213.67 ± 8.14 ^f^	2366.33 ± 32.08 ^c^	3186.33 ± 56.59 ^f^	972.67 ± 53.79 ^c^	79.38 ± 0.05 ^f^
CF-15%	4250.00 ± 37.4 ^d^	2170.67 ± 55.15 ^f^	2079.33 ± 18.01 ^d^	2947.66 ± 49.97 ^g^	777.00 ± 82.27 ^d^	80.20 ± 0.04 ^e^
RF	4021.67 ± 41.88 ^e^	2947.00 ± 27.22 ^a^	1074.67 ± 19.01 ^e^	5095.66 ± 20.59 ^a^	2148.67 ± 25.16 ^a^	85.12 ± 0.06 ^d^
RF-5%	3583.67 ± 26.35 ^f^	2610.67 ± 21.39 ^c^	973.00 ± 17.69 ^f^	4693.00 ± 1.73 ^b^	2082.33 ± 20.50 ^a^	86.95 ± 0.02 ^c^
RF-10%	3441.33 ± 55.64 ^g^	2504.33 ± 14.36 ^d^	937.00 ± 50.59 ^fg^	4600.00 ± 125.37 ^b^	2095.67 ± 119.09 ^a^	87.69 ± 0.29 ^b^
RF-15%	3115.33 ± 41.55 ^h^	2207.33 ± 54.63 ^f^	908.00 ± 18.08 ^fg^	3855.67 ± 61.85 ^c^	1648.33 ± 113.01 ^b^	89.70 ± 0.18 ^a^

The values are presented as mean ± standard deviation (*n* = 3). Superscripts ^a–i^ indicate that the means in the same column are significantly different (*p* < 0.05). CP, edible cactus powder; CF, cassava flour; RF, rice flour. Furthermore, 5%, 10%, and 15% indicate the levels of edible cactus powder added to the cassava flour or rice flour.

**Table 5:** Freeze–thaw stability of cassava flour and rice flour fortified with edible cactus powder.

Sample	Cycle 1 (%)	Cycle 3 (%)	Cycle 5 (%)
CP	45.02 ± 0.61 ^e^	55.07 ± 0.19 ^e^	66.43 ± 0.27 ^e^
CF	26.33 ± 0.25 ^f^	28.07 ± 0.18 ^f^	44.96 ± 0.02 ^f^
CF-5%	24.18 ± 0.31 ^g^	26.69 ± 0.17 ^g^	40.13 ± 0.79 ^g^
CF-10%	21.08 ± 0.33 ^h^	24.12 ± 0.21 ^h^	35.15 ± 0.26 ^h^
CF-15%	19.15 ± 0.27 ^i^	22.32 ± 0.13 ^i^	31.89 ± 0.58 ^i^
RF	65.58 ± 1.74 ^a^	70.56 ± 0.36 ^a^	76.79 ± 0.21 ^a^
RF-5%	55.40 ± 0.46 ^b^	67.61 ± 0.14 ^b^	74.55 ± 0.02 ^b^
RF-10%	52.51 ± 0.29 ^c^	66.19 ± 0.24 ^c^	73.32 ± 0.08 ^c^
RF-15%	50.13 ± 0.09 ^d^	64.93 ± 0.52 ^d^	70.08 ± 0.27 ^d^

The values are presented as mean ± standard deviation (*n* = 3). Superscripts ^a–i^ indicate that the means in the same column are significantly different (*p* < 0.05). CP, edible cactus powder; CF, cassava flour; RF, rice flour. Furthermore, 5%, 10%, and 15% indicate the levels of edible cactus powder added to the cassava flour or rice flour.

**Table 6:** In vitro starch digestibility of cassava flour and rice flour fortified with edible cactus powder.

Sample	RDS (%)	SDS (%)	RS (%)
CP	47.19 ± 0.18 ^d^	21.12 ± 0.01 ^b^	31.67 ± 0.18 ^f^
CF	56.94 ± 1.04 ^b^	6.80 ± 0.60 ^e^	36.25 ± 0.46 ^c^
CF-5%	36.03 ± 0.13 ^f^	12.88 ± 0.61 ^d^	51.08 ± 0.73 ^a^
CF-10%	35.12 ± 0.38 ^f^	16.05 ± 0.03 ^d^	48.82 ± 0.35 ^b^
CF-15%	32.45 ± 0.17 ^g^	18.80 ± 0.7 ^c^	48.74 ± 0.86 ^b^
RF	65.78 ± 0.43 ^a^	6.83 ± 0.71 ^e^	27.37 ± 0.32 ^e^
RF-5%	53.06 ± 0.63 ^c^	12.10 ± 1.21 ^d^	34.83 ± 0.65 ^d^
RF-10%	42.53 ± 0.01 ^e^	21.57 ± 0.01 ^b^	35.88 ± 0.02 ^cd^
RF-15%	32.63 ± 0.95 ^g^	31.02 ± 1.16 ^a^	36.34 ± 1.58 ^c^

The values are presented as mean ± standard deviation (*n* = 3). Superscripts ^a–g^ indicate that the means in the same column are significantly different (*p* < 0.05). CP, edible cactus powder; CF, cassava flour; RF, rice flour. Furthermore, 5%, 10%, and 15% indicate the levels of edible cactus powder added to the cassava flour or rice flour. RDS, rapidly digestible starch; SDS, slowly digestible starch; RS, resistant starch. RDS, SDS, and RS are expressed as percentages of total starch.

## Data Availability

The original contributions presented in this study are included in the article. Further inquiries can be directed to the corresponding authors.
